# Changes in the Expression of Vascular Endothelial Growth Factor after Fetal Tracheal Occlusion in an Experimental Model of Congenital Diaphragmatic Hernia

**DOI:** 10.1155/2013/958078

**Published:** 2013-01-27

**Authors:** E. Sanz-López, E. Maderuelo, D. Peláez, P. Chimenti, R. Lorente, M. A. Muñoz, M. Sánchez-Luna

**Affiliations:** ^1^Neonatology Service, Hospital General Universitario Gregorio Marañón, Calle Doctor Esquerdo 46, 28007 Madrid, Spain; ^2^Pediatric Surgery Service, Hospital General Universitario Gregorio Marañón, Madrid, Spain; ^3^Immunology Service, Hospital General Universitario Gregorio Marañón, Madrid, Spain

## Abstract

*Introduction*. Vascular endothelial growth factor (VEGF), an angiogenic factor secreted by type II pneumocytes, could play a role in congenital diaphragmatic hernia (CDH) pathogenesis. Animal studies suggest that VEGF accelerates lung growth. *Aim*. To quantify VEGF on fetal lungs in a nitrofen rat model for CDH and to analyze the effect of tracheal occlusion (TO) in VEGF in fetal lung rats after nitrofen and in control rats not exposed to nitrofen. *Methods*. Pregnant rats received nitrofen on day 9.5 of gestation. Fetuses were divided into 2 groups: those that underwent TO on day 20 and those that did not. On day 21, fetuses were delivered, and the lungs were dissected for subsequent VEGF quantification. *Results*. CDH was detected in 43% of the fetuses that received nitrofen. Fetuses with CDH showed significantly reduced lung weight/fetal weight ratio and lower VEGF levels than the remainder. A higher VEGF value was observed after TO. *Conclusions*. VEGF protein was significantly lower in fetuses with CDH. TO induced a significant increase in VEGF compared to the fetuses that did not undergo TO. Although not statistically significant, we observed higher VEGF levels in fetuses with CDH and TO compared to fetuses with CDH and no further intervention.

## 1. Introduction

Congenital diaphragmatic hernia (CDH) is a malformation associated with incomplete closure of the pleuroperitoneal membrane, secondary herniation pushing the abdominal viscera into the thorax, and pulmonary hypoplasia. The patient usually experiences severe respiratory failure and pulmonary hypertension resulting from pulmonary hypoplasia. Consequently, morbidity and mortality are high. The prevalence of CDH is estimated in 1 case for every 3000 newborns [1], although it is difficult to measure given the high intrauterine mortality (spontaneous and induced). According to the Congenital Diaphragmatic Hernia Registry, which pools data from more than 50 centers, survival ranges from 50% to 67% depending on the series [2–4].

Current investigations are aimed at prevention and the search for an effective treatment for pulmonary hypoplasia. Several strategies have been proposed to improve growth of the hypoplastic lung before birth, the most outstanding being occlusion of the fetal trachea, which has been shown to stimulate growth of the fetal lung in an experimental model [5]. 

Adequate fetal pulmonary vascularization is an essential component to the development of a mature alveolus that is capable of performing effective gas exchange after birth [6]. Angiogenesis requires several growth factors, mainly vascular endothelial growth factor (VEGF), a potent angiogenic factor secreted by type II pneumocytes in the developing lung that mediates vasculogenesis and postnatal vascular remodelling. VEGF could play a role in the pathogenesis of CDH, and recent studies in rodents suggest that it could accelerate growth in prenatal nitrofen-induced hypoplastic lungs [6, 7]. 

## 2. Objectives

The objective of this paper was to measure VEGF in the lungs of fetuses with CDH induced by administration of nitrofen to the mother, and to analyze the effect in VEGF values after occlusion of the fetal trachea in an experimental model of CDH in rat fetuses.

## 3. Material and Methods

The study protocol was approved by the Animal Investigation Committee of Hospital Gregorio Marañón. All procedures were performed according to European legislation for the protection of animals used for scientific purposes (Directive 86/609/EEC).

### 3.1. Study Subjects

 The study subjects were female Sprague-Dawley rats weighing 225–250 g and male rats of proven fertility. The animals were housed in custom facilities at the research laboratory at 55% humidity and 21°C. They were exposed to a 12/12-hour light-dark cycle and received special granulated feed and water on demand. Surgical procedures were performed in rooms equipped with the necessary specific equipment.

### 3.2. Experimental Model

#### 3.2.1. Controlled Fertilization

 After 24 hours' visual and olfactory contact, the females were cohoused at 20:00 hours with a male at a ratio of 3 : 1 so that they could be fertilized during the night. Day 0 started at 00:00 hours on the day cytology demonstrated the presence of a sperm plug in the vagina of the fertilized female.

#### 3.2.2. Administration of Nitrofen

On day 9.5 of gestation, the pregnant rats received 100 mg of nitrofen (2, 4-dichlorophenyl 4-nitrophenyl ether diluted in 2 mL of olive oil) through an orogastric tube. The control animals received the same volume of olive oil without nitrofen.

#### 3.2.3. Tracheal Occlusion

**Figure 1:**
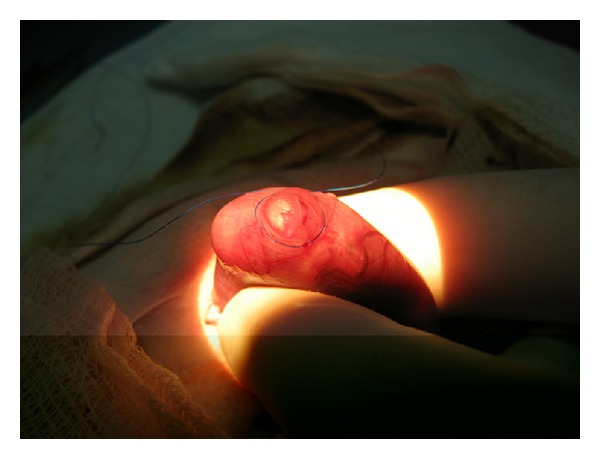
Exposure of the fetal head.

**Figure 2:**
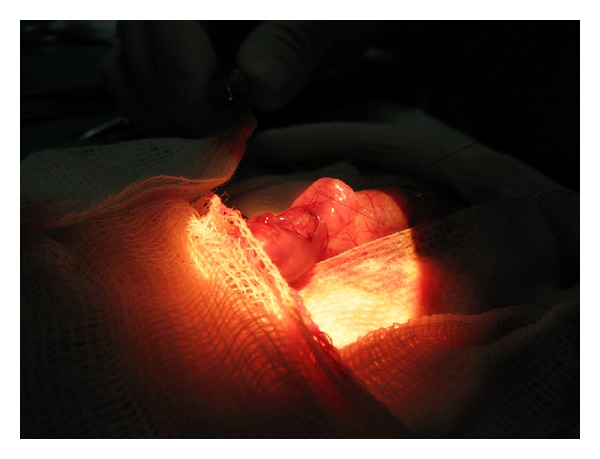
Intrauterine tracheal occlusion.

The trachea of the fetuses was occluded on day 20 of gestation. The pregnant rats were anesthetized with inhaled 3% isoflurane and 1.5% maintenance isoflurane. Intramuscular ketorolac was used as a postoperative analgesic. Rectal temperature was monitored constantly and maintained using a homeothermic blanket for rats. Access to the abdominal cavity was by medial minilaparotomy, which enabled the uterine horns to be visualized. Once the uterine wall was opened, the fetal head and neck were exposed (Figure 1), using a surgical microscope (Zeiss OPMI 99, Zeiss Inc. Oberkochen, Germany) to facilitate the maneuvers. The fetal neck was exposed and hyperextended, and a small transverse medial incision was done. The trachea was exposed, and a nonabsorbable 10/10 nylon ligament was tied to close the trachea (Figure 2). The occlusion was confirmed by direct visualization, and the fetus was returned to the uterus. Each mother underwent a number of hysterotomies that varied according to the number of fetuses, surgical time required, difficulty of the technique for each subject, and estimated time of temperature loss. In all cases, the objective was to perform 6 hysterotomies with tracheal occlusion in half of the fetuses and exposure with no manipulation of the trachea in the remainder (tracheal occlusion control group). The maternal laparotomy was closed on 2 planes (muscle aponeurosis and skin). Recovery was confirmed by evaluating normal activity and recovery of appetite and intestinal transit.

#### 3.2.4. Extraction of the Fetal Lungs

The anesthetized rat underwent Cesarean section on day 21 (term was on day 22). The rat and her fetuses were then sacrificed. After weighing the fetus, the presence or absence of a diaphragmatic defect was confirmed, and 2 lung explants were taken and weighed before being frozen immediately in liquid nitrogen at –80°C for subsequent measurement of VEGF using immunoanalysis.

### 3.3. Statistical Analysis

All results are expressed as mean ± SD. The mean fetal weight, lung weight, lung weight/fetal weight ratio, and VEGF were calculated using SPSS 16.0 and compared between groups using Student's *t*-test. Statistical significance was defined as *P* < 0.05.

## 4. Results

137 fetuses were analyzed and divided into groups as follows: Group 1 comprised 62 control fetuses and Group 2 comprised 75 fetuses exposed to nitrofen. A defect in the diaphragm was observed in 32 of the fetuses exposed to nitrofen (42.6%). No cases of diaphragmatic hernia were observed in the control group.

Mean fetal weight, mean lung weight, and the ratio of lung weight to fetal weight were measured and compared between groups. The mean weights of the fetuses and lungs were significantly lower in Group 2, although no significant differences were found in the ratio of lung weight to fetal weight between the groups (Table 1).

In Group 2, fetuses who developed CDH had a significantly lower lung weight and lower ratio of lung weight to fetal weight ratio than those who did not (Table 2).

A third analysis of weight in the Group 2 fetuses with CDH (32 fetuses) compared the results to those fetuses that underwent tracheal occlusion (6 cases) and those that did not (26 fetuses) (Table 3). Statistically significant differences were found between the groups in mean fetal weight, but not in lung weight (*P* = 0.08) or in the ratio of lung weight to fetal weight.

**Figure 3:**
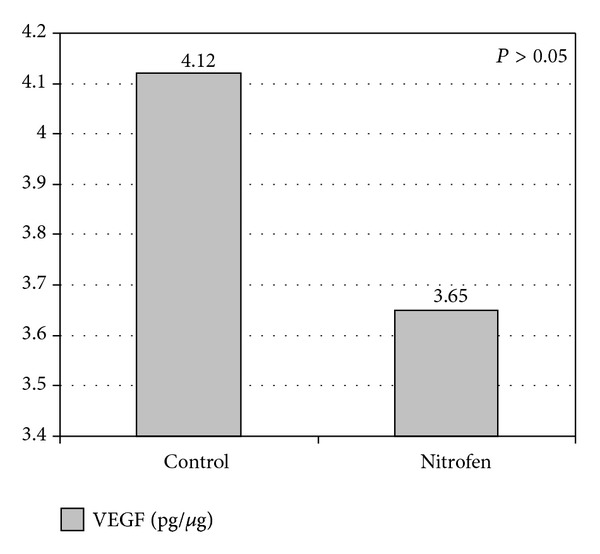
Vascular endothelial growth factor (VEGF) in Group 1 (controls) versus Group 2 (nitrofen). Values are expressed as mean (pg/*μ*g).

**Figure 4:**
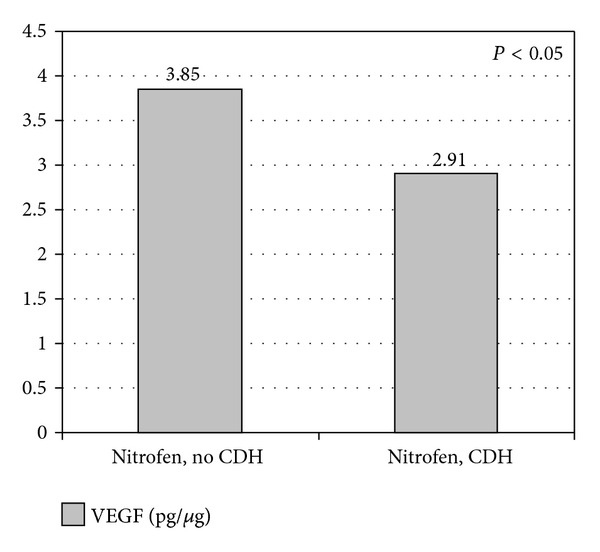
Vascular endothelial growth factor VEGF in Group 2 (nitrofen fetuses). Comparing fetuses with and without CDH. Values are expressed as mean in pg/*μ*g.

The difference in the mean VEGF value between Group 1 and Group 2 was not statistically significant (4.12 ± 0.60 and 3.65 ± 0.74 pg/*μ*g; *P* = 0.157) as seen in Figure 3. In Group 2, the VEGF value was significantly lower in the fetuses that had CDH than in those that did not (2.91 ± 0.59 pg/*μ*g versus 3.85 ± 0.70 pg/*μ*g; *P* = 0.02) (Figure 4). 

**Figure 5:**
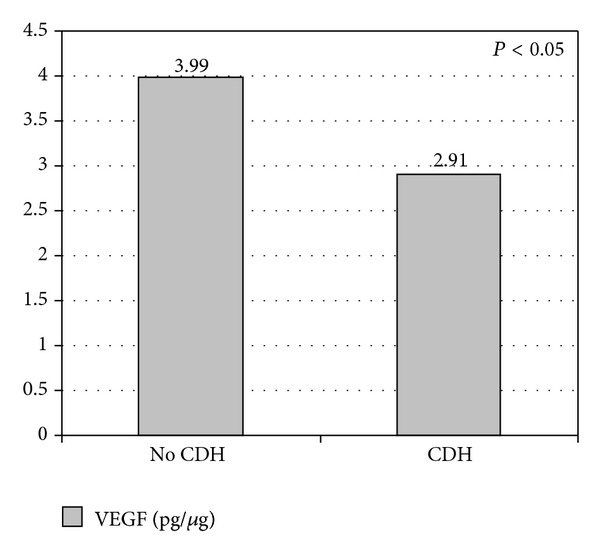
Vascular endothelial growth factor VEGF in fetuses with and without congenital diaphragmatic hernia. Values are expressed as mean in pg/*μ*g.

Comparing the subgroup of fetuses who developed CDH with the remaining fetuses who did not develop CDH (Group 1 + Group 2 without CDH), the VEGF values were significantly lower in fetuses with CDH than in the other fetuses (2.91 ± 0.59 pg/*μ*g versus 3.99 ± 0.73 pg/*μ*g; *P* = 0.03) (Figure 5).

**Figure 6:**
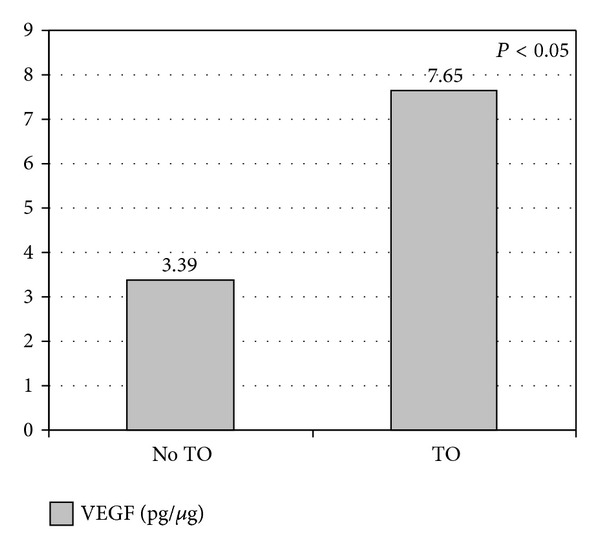
Vascular endothelial growth factor, VEGF, in fetuses with congenital diaphragmatic hernia. Comparison of tracheal occlusion versus no tracheal occlusion. Values are expressed as mean in pg/*μ*g.

The mean VEGF value of the fetuses that underwent tracheal occlusion was 7.65 ± 0.92 pg/*μ*g compared with 3.39 ± 0.60 pg/*μ*g in the total of fetuses that did not undergo tracheal occlusion (*P* = 0.00) (Figure 6).

**Figure 7:**
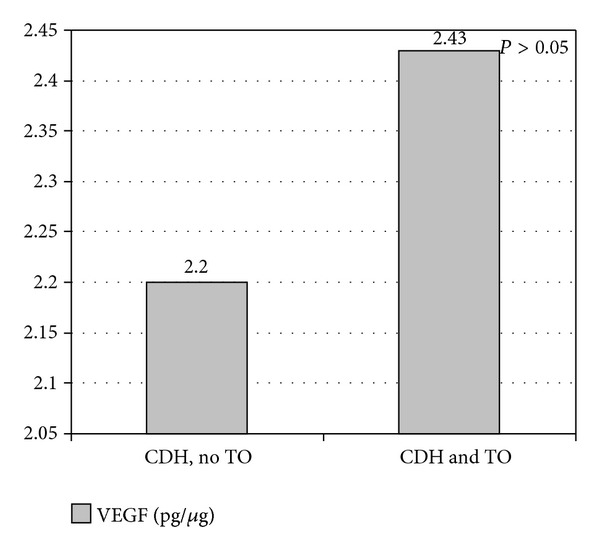
Vascular endothelial growth factor (VEGF) in fetuses with congenital diaphragmatic hernia (CDH). Comparison of tracheal occlusion (TO) versus no TO. Values are expressed as mean (pg/*μ*g).

**Figure 8:**
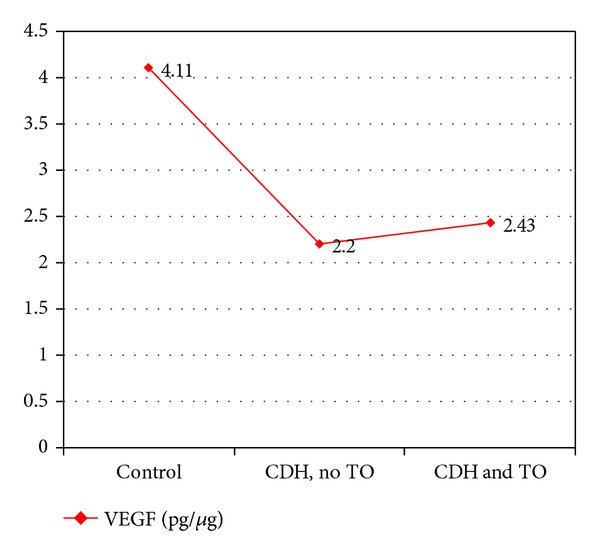
VEGF values in control rats, fetuses with congenital diaphragmatic hernia (CDH), and fetuses with CDH that underwent tracheal occlusion (TO).

Finally, we compared the VEGF values of fetuses with CDH that underwent tracheal occlusion with those that did not and found that the differences were not statistically significant (2.43 ± 0.66 pg/*μ*g versus 2.20 ± 0.81 pg/*μ*g; *P* = 0.27) (Figures 7 and 8).

## 5. Discussion

### 5.1. Experimental Model

Experimental induction of diaphragmatic defects in rats using nitrofen is a predictable and easily reproducible approach for the study of CDH. The fetuses develop CDH at a specific point during gestation once the correct dose is administered [7]. Nitrofen-induced CDH is associated with malformations that are similar to the human CDH phenotype, for example, pulmonary hypoplasia, neural crest defects, cardiovascular defects, and other conditions [8–10]. Moreover, a phenotype similar to that of Fryns syndrome in humans has been reported in rats [11]. Experimental studies show that passage of the abdominal viscera into the thorax through a defect in the diaphragm is independent of the development of pulmonary hypoplasia. In the nitrofen model, pulmonary hypoplasia does not only result from diaphragmatic hernia and direct compression of the lung, since pulmonary hypoplasia is constant, whereas only 40–80% of fetuses develop CDH. These results were corroborated in our study, in which we found a lower lung weight in fetuses exposed to nitrofen than in control fetuses (Table 1). However, the ratio of lung weight to body weight was significantly lower in those fetuses that developed CDH. These findings could enable us to act against the mechanisms that govern lung development, regardless of the development of hernia.

### 5.2. Role of VEGF

VEGF is an angiogenic factor secreted by type II pneumocytes that induces growth in endothelial cells *in vitro*, angiogenesis *in vivo*, and proliferation of epithelial cells in the lungs. VEGF plays a crucial role in the development of the human fetal lung. Expression increases between the canalicular and saccular phases, reaching a peak at week 31 of gestation. It subsequently decreases during the alveolar phase, thus acquiring a key role in alveolar development. Expression in the rat fetal lung peaks on day 16 of gestation, at the beginning of the saccular phase and before closure of the diaphragm [12, 13].

Various experimental findings suggest that VEGF plays an important role in pulmonary morphogenesis and in the pathogenesis of CDH [6, 14]; however, few data are available on the role of VEGF in the pathogenesis of CDH in humans. Increased expression of VEGF in small lung arteries and supernumerary arteries has been observed in newborns with pulmonary hypertension who died of CDH and may represent an attempt by the fetus to stimulate angiogenesis in lungs in which development has stopped [15]. These data differ from those obtained in experimental CDH models, in which the amount of VEGF is reduced, possibly because the method used to measure protein values varies between studies [16]. Our calculation of VEGF values in the fetal lung (measured using immunoanalysis and expressed as pg/*μ*g) revealed no differences in VEGF levels between the fetuses that received nitrofen and the control group. In contrast, we did find a statistically significant difference in VEGF levels between fetuses with CDH and fetuses without it, as well as in the group of fetuses that received nitrofen. Consistent with the findings of other studies, our results confirm the importance of VEGF in lung morphogenesis and suggest a role for VEGF in the pathogenesis of CDH [6, 14].

The mechanical distension produced by tracheal occlusion accelerates growth and maturation of the lung; however, it delays differentiation of type II pneumocytes and formation of surfactant [17]. These mechanical factors seem to affect expression of VEGF in lung tissue.  Figure 7 shows the trend toward recovery of VEGF levels in fetuses with CDH that underwent tracheal occlusion in our sample, although the differences were not statistically significant. Recent reports indicate that the increase in lung volume and the ratio of lung weight to fetal weight are greater when tracheal occlusion is prolonged [18, 19].

In summary, in this animal model of CDH in fetal rats induced by nitrofen administrated to pregnant rats, lung VEGF values were significantly lower in fetuses with CDH compared to those who did not develop CDH. Tracheal occlusion induced a significant increase in the mean VEGF compared to the total of fetuses that did not undergo tracheal occlusion, although differences were not statistically significant we did observe a trend toward reduced expression of this protein in fetuses with CDH. Tracheal occlusion could improve expression of VEGF in the lungs. 

## Figures and Tables

**Table 1:** Comparison of mean weight by group (control versus nitrofen).

	Control (*n* = 62)	Nitrofen (*n* = 75)	*P *
Fetal weight (mean)	5.16 g ± 0.41	4.42 g ± 0.71	**0.00**
Lung weight (mean)	0.057 g ± 0.03	0.049 g ± 0.02	**0.03**
Lung weight/fetal weight	0.011 ± 0.01	0.010 ± 0.02	0.96

**Table 2:** Group exposed to nitrofen (75 fetuses). Comparison of mean weights according to presence or not of congenital diaphragmatic hernia (CDH).

	No CDH (*n* = 43)	CDH (*n* = 32)	*P *
Fetal weight (mean)	4.44 g ± 0.70	4.36 g ± 0.73	0.58
Lung weight (mean)	0.053 g ± 0.05	0.039 g ± 0.02	**0.00**
Lung weight/fetal weight	0.0119 ± 0.01	0.0071 ± 0.01	**0.00**

**Table 3:** Fetal weight, lung weight, and lung-to-fetal ratio of fetuses with CDH divided into two groups—tracheal occlusion and no tracheal occlusion.

	Tracheal occlusion (*n* = 6)	No tracheal occlusion (*n* = 26)	*P *
Fetal weight (mean)	3.40 g ± 0.28	4.46 g ± 0.29	**0.01**
Lung weight (mean)	0.020 g ± 0.04	0.031 g ± 0.06	0.08
Lung weight/fetal weight	0.0061 ± 0.00	0.0072 ± 0.06	0.50
